# Polymorphism Patterns and Socioeconomic Characteristics and Their Influence on the Risk of Preeclampsia

**DOI:** 10.3390/medicina60060890

**Published:** 2024-05-28

**Authors:** Flavius George Socol, Marius Craina, Simona-Alina Abu-Awwad, Ioana Denisa Socol, Simona Sorina Farcas, Ahmed Abu-Awwad, Denis Serban, Adina-Ioana Bucur, Elena Bernad, Lioara Boscu, Laura Claudia Popa, Nicoleta Ioana Andreescu

**Affiliations:** 1 Doctoral School, University of Medicine and Pharmacy “Victor Babeş”, Eftimie Murgu Sq. No. 2, 300041 Timisoara, Romania; george.socol@umft.ro (F.G.S.); ioana.socol@umft.ro (I.D.S.); lioara.boscu@umft.ro (L.B.); 2 Ist Clinic of Obstetrics and Gynecology, “Pius Brinzeu” County Clinical Emergency Hospital, 300723 Timisoara, Romania; mariuscraina@hotmail.com (M.C.); alina.abuawwad@umft.ro (S.-A.A.-A.); bernad.elena@umft.ro (E.B.); 3 Department of Obstetrics and Gynecology, Faculty of Medicine, “Victor Babes” University of Medicine and Pharmacy, 300041 Timisoara, Romania; denis.serban@umft.ro; 4 Center for Laparoscopy, Laparoscopic Surgery and In Vitro Fertilization, “Victor Babes” University of Medicine and Pharmacy, 300041 Timisoara, Romania; 5 Department of Microscopic Morphology—Genetics, Center of Genomic Medicine, University of Medicine and Pharmacy “Victor Babes”, Eftimie Murgu Sq. No. 2, 300041 Timisoara, Romania; farcas.simona@umft.ro (S.S.F.); laura.popa@umft.ro (L.C.P.); andreescu.nicoleta@umft.ro (N.I.A.); 6 Department XV—Discipline of Orthopedics—Traumatology, “Victor Babes” University of Medicine and 21 Pharmacy, 300041 Timisoara, Romania; ahm.abuawwad@umft.ro; 7 Research Center University Professor Doctor Teodor Sora, “Victor Babes” University of Medicine and 23 Pharmacy, 300041 Timisoara, Romania; 8 Institute of Cardiovascular Diseases Timisoara, 300310 Timisoara, Romania; 9 Department of Functional Sciences, Faculty of Medicine, “Victor Babes” University of Medicine and Pharmacy from Timișoara, 300041 Timișoara, Romania

**Keywords:** preeclampsia, genetic polymorphisms, ACE gene, rs4343 polymorphism, genetic predisposition, personalized management, pregnancy complications

## Abstract

*Background*: Preeclampsia (PE) is a critical condition affecting pregnancies worldwide. Understanding its etiology, particularly the genetic factors, is vital. This study aims to investigate the association between ACE gene polymorphisms, specifically the ACE G2350A (rs4343) variant, and the predisposition to PE, offering insights into the genetic predisposition towards this complex condition. *Methods*: A case-control study was conducted with 140 participants without PE (Control Group) and 128 participants diagnosed with PE (PE Group). The study focused on comparing the prevalence of the rs4343 polymorphism between the groups. *Results*: The analysis identified a significantly reduced risk associated with the AG genotype and an insignificant increase in risk with the AA genotype. Statistically significant differences in demographic and clinical characteristics, such as BMI and marital status, were observed between the groups, suggesting a multifaceted risk profile for PE that includes genetic, environmental, and socio-economic factors. *Conclusions*: The study highlight the significant role of genetic variations, specifically the ACE G2350A (rs4343) polymorphism, in influencing PE predisposition. It highlights the intricate interplay between genetic predispositions and other risk factors in the development of PE. Further research is encouraged to expand on these findings and explore a wider range of genetic polymorphisms and their interactions with environmental factors.

## 1. Introduction

Preeclampsia (PE), a complex condition impacting multiple bodily systems, occurs in 3–8% of pregnancies within the United States and between 1.5% and 16.7% globally. This disorder is responsible for 60.000 maternal deaths and over 500,000 cases of pre-term births across the world annually [1]. 

PE is clinically identified by elevated blood pressure readings—specifically, a systolic blood pressure (SBP) of 140 mm Hg or higher, or a diastolic blood pressure (DBP) of 90 mm Hg or more. This condition also presents with significant protein levels in the urine, exceeding 300 mg over 24 h, and becomes diagnosable after the 20th week of pregnancy. Beyond these primary indicators, individuals with PE may experience a variety of other symptoms. These can include edema often noticeable in the hands, feet, and face; visual disturbances such as blurred vision or light sensitivity; severe headaches; abdominal pain, particularly in the upper right side; and thrombocytopenia. These symptoms highlight the complex and multisystemic nature of PE, affecting various parts of the body and necessitating comprehensive medical attention to manage both maternal and fetal risks [2]. In the absence of timely and effective intervention, individuals suffering from PE are significantly more susceptible to a range of critical health issues. These include life-threatening conditions such as the rupture of the liver and failure of the kidneys. Moreover, this condition significantly increases the likelihood of encountering unfavorable outcomes for the newborn, including, but not limited to, low birth weight and neonatal asphyxia, a condition in which the child does not receive enough oxygen at birth. These adverse effects highlight the urgent need for early detection and management of PE to protect the health of both the mother and the child [3].

The underlying mechanisms leading to PE are complex and remain not entirely deciphered. Inadequate formation of the placental tissue, known as poor placentation, followed by reduced blood flow to the placenta, or placental ischemia, is strongly implicated as a key factor in its development [4]. These issues are thought to be connected to malfunctions within the renin–angiotensin system (RAS) [5]. The RAS in the uterus and placenta, which operates differently from the RAS associated with the kidneys, plays a pivotal role in managing blood flow to the placenta. When abnormalities occur within this uteroplacental RAS, it can lead to disrupted blood supply and contribute to the adverse changes observed in PE, such as hypertension and damage to other organs [6]. Further research into PE reveals that patients with this condition show distinctive patterns of RAS activity compared to healthy individuals. Notably, these patterns include reduced levels of renin and angiotensin II, which are vital components of the RAS. However, an increased sensitivity to angiotensin II could amplify the risk of high blood pressure [7]. This altered response in the RAS suggests a complex interplay of factors that exacerbate the symptoms of PE, highlighting the need for a deeper understanding of these mechanisms to develop more effective treatments. 

One genetic case-control study has identified potential links between PE and various genetic polymorphisms, highlighting the complex interplay between genetics and the disease’s development [8]. However, the challenge of reproducing these findings across different studies introduces uncertainty regarding the specific genes that play pivotal roles in the pathogenesis of PE [9]. This inconsistency highlights the complexity of genetic influences on the disease and the need for further research to clarify these associations. The angiotensin-converting enzyme (ACE), a critical element of the RAS, converts angiotensin I, an inactive form, into angiotensin-sin II, its active counterpart [10]. Located on chromosome 17q23, the ACE gene presents numerous polymorphisms, including the ACE G2350A (rs4343), which influence the enzyme’s activity [11].

These genetic variations are at the forefront of research into the etiology of PE, suggesting that a genetic predisposition, especially related to components of the RAS, could significantly influence an individual’s risk of developing the condition. The exploration of these genetic connections opens up new avenues for understanding the inherited aspects of PE, offering the potential for targeted interventions and personalized treatment approaches based on an individual’s genetic makeup.

The primary goal of the study described is to investigate the association between different genotypes (GG, AG, AA) and the risk of PE in two distinct groups: a control group (group 1) and a group with PE (group 2). By comparing the frequency of these genotypes between the two groups and calculating the Odds Ratio (OR) for each genotype, the study aims to determine whether certain genotypes are associated with a higher or lower risk of developing PE. Thus, this study could contribute to understanding the genetic mechanisms underlying PE, offering potential pathways for identifying women at high risk and developing more effective prevention or treatment strategies.

## 2. Materials and Methods

### 2.1. Study Design and Population

This research was structured as a case-control study, aimed at uncovering the genetic foundations of preeclampsia (PE) by comparing the prevalence of specific genetic polymorphisms between patients diagnosed with PE and those who had a pregnancy without pathologies. Patients were monitored and admitted for delivery at the Department of Obstetrics and Gynecology of the “Pius Brînzeu” Clinical Emergency Hospital in Timișoara, Romania. The duration of the study was 4 years. The women included in the study were those admitted for both delivery and obstetric complications, which resulted in childbirth. Out of 3653 pregnant patients admitted to this clinic, 268 were included in the study based on strict inclusion and exclusion criteria. These were divided into two main cohorts: Group 1, consisting of 140 pregnant women without PE (control group), and Group 2, consisting of 128 pregnant women diagnosed with PE (PE group). The medical data and blood samples presented in this study were collected from the patients’ observation records compiled at the time of hospital admission for delivery. All women diagnosed with PE within our cohort were approached for participation in the study. Out of these, a total of 128 women consented and met the inclusion criteria. These women were continuously monitored and documented, ensuring comprehensive data collection.

Controls were selected using a rigorous matching process to minimize bias. Specifically, for each woman in the PE group, the next woman admitted to the clinic who met the control criteria (age, gestational age, absence of PE, and other exclusion conditions) was selected, ensuring a comparable baseline.

The discrepancy in the number of controls versus cases is attributed to the strict matching criteria and high variability inpatient admissions. Despite thorough efforts, it was not always feasible to find an exact match for every case, leading to slightly fewer controls. This approach, however, was essential to maintain the integrity and specificity of the control group, ensuring that only those women who closely matched the cases on critical variables were included.

### 2.2. Inclusion and Exclusion Criteria

To maintain the integrity and objectivity of the study’s outcomes, we have meticulously defined precise criteria for participant inclusion and exclusion, aimed at mitigating the influence of extraneous variables.

Inclusion criteria includes pregnant women aged between 18 and 45 years with a documented diagnosis of PE, confirmed singleton pregnancy by ultrasound to avoid confounding effects of multiple pregnancies, and no family history of PE suggesting potential genetic predisposition. Participants must be willing to undergo genetic testing and provide DNA samples to identify polymorphisms associated with PE. They should have detailed prenatal care records, including regular blood pressure and urine protein measurements, and no history of smoking, alcohol, or substance abuse during pregnancy. Additionally, participants must agree to provide access to their medical records for a comprehensive review of their pregnancy history and outcomes and to participate in postpartum follow-up studies to assess long-term health outcomes related to PE, including cardiovascular health.

Exclusion criteria include the presence of any pre-existing chronic conditions such as diabetes mellitus, chronic hypertension, or renal diseases that could affect pregnancy progression or study outcomes. Pregnant women who have undergone major surgical procedures during pregnancy or used unapproved medications or supplements that could influence study results are excluded. Additionally, a history of genetic or chromosomal disorders in the extended family that could impact study outcomes was specified as an exclusion criterion. This includes any diagnosed conditions such as Down syndrome, Turner syndrome, cystic fibrosis, hemophilia, or any other inheritable genetic disorders known to influence pregnancy outcomes or maternal health. Detailed family medical histories were reviewed to identify any presence of these disorders. This precaution ensures the integrity of the study by excluding participants whose genetic predispositions could confound the results, providing a clearer understanding of the association between the specific genetic polymorphisms under investigation and the risk of developing preeclampsia. Severe infections or communicable diseases during pregnancy, and exposure to radiation or known pregnancy-affecting toxic substances are also grounds for exclusion. Finally, pregnancies resulting from assisted reproductive technologies are excluded due to the additional variables involved in these cases. Additionally, due to the well-known high risk of preeclampsia in obese women, it was decided to exclude all women with a BMI greater than 30 kg/m^2^ from the study.

### 2.3. Genetic Screening and Polymorphism Analysis

We collected whole blood samples in EDTA tubes from all subjects and the genomic DNA was extracted by using the Genomic DNA Whole Blood Kit (Code 102 MagCore^®^) on a MagCore^®^ Plus II instrument (RBC Bioscience, New Taipei City, Taiwan), according to the manufacturer’s protocol. The quantity and quality of DNA samples were measured by BioTek Epoch microplate spectrophotometer (Agilent Technologies, Santa Clara, CA, USA) and then the samples were diluted to 10 ng/μL. The genotyping reaction mix contained 10 μL of TaqMan Genotyping Master Mix, 0.5 μL of TaqMan^®^ SNP Genotyping Assay (C_11942562_20), 8.5 μL nuclease-free water and 1 μL DNA template. The experiments were run on an Applied Biosystems QuantStudio 7 Flex (Thermo Fisher Scientific, Carlsbad, CA, USA). The allelic discrimination data were plotted as a comparison of allele 1 (VIC dye) and allele 2 (FAM dye) using real-time PCR instrument software (QuantStudio Real-Time PCR Software v1.3).

### 2.4. Statistical Analysis

The statistical analysis of our study was conducted using MedCalc [12] and GraphPad Prism 6, two specialized statistical software packages known for their advanced capabilities in biomedical research. This allowed for a precise evaluation of our data. Our statistical methodology included both the Z-test and the T-test. The Z-test was applied to compare the proportions of categorical variables between two independent groups, providing insights into differences in genotype frequencies. Meanwhile, the T-test was used to compare the means of continuous variables, facilitating an understanding of any significant disparities in physiological measures related to PE among different genotypes.

Statistical significance was determined using a *p*-value threshold of less than 0.05. This criterion was applied across all statistical tests to identify meaningful associations and differences within our data set. The use of SPSS software (IBM SPSS Statistics for Windows, Version 25.0. IBM Corp: Armonk, NY, USA) further complemented our analysis, providing a robust platform for data handling and complex statistical calculations. Odds Ratios (ORs) with 95% Confidence Intervals (CIs) were calculated to assess the association between the identified genotypes (GG, AG, AA) and the risk of PE. The ORs provided a measure of the likelihood of developing PE for each genotype compared to the reference group.

### 2.5. Ethical Considerations

In compliance with ethical standards, each participant in this research provided their informed consent voluntarily before their inclusion. This consent encompassed their willingness to partake in the research activities, permission for the collection of their blood samples for scientific analysis, and approval for the use of their personal and health-related data. The thoroughness of this consent procedure was vital for respecting the participants’ autonomy, and ensuring their rights and preferences were honored. 

Additionally, the study implemented stringent measures to protect the confidentiality and privacy of all participants. Every piece of data collected during the research was meticulously anonymized to remove any personal identifiers, reflecting the study’s dedication to upholding the highest ethical principles. Ethical approval for this study was granted by the ethics committee of “Pius Brînzeu” Clinical Emergency Hospital in Timișoara, Romania, under approval number 72 dated 28 June 2021, highlighting its adherence to ethical research practices.

## 3. Results

Comparative analysis outlines the demographic and clinical characteristics of participants. (Table 1). The evaluation spans various dimensions including age, days of hospitalization, Body Mass Index (BMI), gravidity, marital status, education level, employment status, and area of residence. Regarding hospitalization days for the two groups, the Control group had an average hospital stay of 3.4 days with a standard deviation of 6.0 days. The PE group had an average stay of 5.0 days with a standard deviation of 7.1 days. The indication for hospitalization was either the onset of labor or an obstetrical complication that led to the termination of the pregnancy through delivery. 

Also, this table shows statistically significant differences between the groups in BMI distribution, marital status, and employment status. The BMI is calculated at the time of delivery, as the pre-pregnancy BMI (specifically those over 30 kg/m^2^ could have influenced the study results, given that obesity is a well-known risk factor for PE/E. No woman with a pre-pregnancy BMI over 30 kg/m^2^ was included in the study. The *p*-values for BMI categories (<25, 25–29.9, ≥30) are <0.0001, 0.0033, and <0.0001, respectively, indicating a strong association with the occurrence of PE. Marital status (married, engaged) shows *p*-values of 0.0434 and 0.0378, respectively, and employment status shows a *p*-value of 0.0115. These findings highlight the significant demographic and clinical differences between the control and PE groups.

Marked differences in vital parameters between the Control Group and the PE Group are evident in Table 2. The PE Group has significantly higher mean systolic blood pressure (SBP) and diastolic blood pressure (DBP) compared to the Control Group. Heart rate is also elevated in the PE Group, while oxygen saturation (SpO2) is slightly lower. These statistically significant differences highlight the substantial physiological impact of PE.

Table 3 compares the control group and the PE group in terms of Apgar scores, gestational age, and neonatal birth weight. The data indicates significant differences between the groups, with the PE group showing lower mean values for Apgar scores, gestation duration, and newborn weight. All differences are statistically significant, as reflected in the respective *p*-values.

The analysis focuses on the distribution of a genetic polymorphism and its association with preeclampsia (PE) between two groups is presented in Table 4. The genotypes considered are GG, AG, and AA. The odds ratios (OR) provide insight into the potential genetic influence on PE predisposition. A significant difference in genotype distribution between the groups is noted, with the GG genotype showing a lower risk, while the AA genotype indicates an increased risk, though not statistically significant. The *p* value of 0.021 highlights the significant genotype distribution differences, underscoring the genetic complexity in PE development.

## 4. Discussion

Preeclampsia (PE) is a condition that complicates a significant portion of pregnancies worldwide, with a notable impact on maternal and neonatal health [13]. A key aspect in the pathophysiology of PE is the dysregulation of the renin–angiotensin system (RAS), which plays an important role in regulating blood pressure and vascular homeostasis during pregnancy. Genetic predispositions, especially within the renin–angiotensin system (RAS), have been implicated in the predisposition to PE, highlighting the complex interplay between genetic factors and the disease’s pathogenesis [14]. Research suggests that genetic variations within RAS genes, such as angiotensin-converting enzyme (ACE), may influence the balance of vasoactive substances, leading to characteristic aberrant vascular responses in PE [15]. Specifically, the ACE G2350A polymorphism, through its impact on ACE enzyme activity, has been linked to altered levels of angiotensin II and bradykinin, key mediators of vascular tone and endothelial function [16]. Additionally, emerging evidence suggests a link between immune dysfunction and the pathogenesis of PE [17]. Dysfunctional maternal immune responses, including aberrant activation of inflammatory pathways and impaired tolerance to fetal antigens, contribute to endothelial dysfunction and placental ischemia, defining features of PE [18]. Genetic factors involved in immune modulation, such as polymorphisms in genes encoding cytokines and human leukocyte antigens (HLA), may predispose individuals to an exaggerated inflammatory response, thereby increasing predisposition to PE [19].

Furthermore, the role of oxidative stress in the development of PE has drawn significant attention. The imbalance between the production of reactive oxygen species (ROS) and antioxidant defense mechanisms leads to endothelial dysfunction, systemic inflammation, and placental oxidative damage, all implicated in the pathophysiology of PE [20]. Genetic variants affecting the activity of antioxidant enzymes, such as superoxide dismutase and glutathione peroxidase, may influence predisposition to oxidative stress-related complications in PE [21].

Despite the identification of various polymorphisms within genes related to the RAS and their associations with hypertension, direct correlations with PE remain underexplored [22]. This gap in knowledge underscores the need for more detailed investigations into the genetic components that may contribute to PE, offering potential avenues for improved risk assessment and targeted interventions.

In this article, we explore the relationship between genetic polymorphisms and the predisposition to PE, through the comparative analysis presented in our results. 

This investigation of the ACE G2350A variants within the study population highlighted the differential distribution of these alleles and their potential correlation with the susceptibility to PE [23]. The findings highlight the important role of these genetic variations in mediating the pathophysiological mechanisms that contribute to the development of PE. This study provides a better understanding of how specific genotypes, particularly the ACE G2350A variants, may influence the biological pathways implicated in the regulation of placental function and maternal vascular response, thereby modulating the risk of PE. The association between these variants and PE risk reinforces the importance of genetic factors in the etiology of this complex disorder, offering potential targets for genetic screening and personalized intervention strategies in the management of PE. The association with the AG genotype suggests potential pathways through which genetic factors may influence the risk of PE, opening avenues for future research into targeted interventions or screening strategies. Conversely, the elevated risk, albeit non-significant, associated with the AA genotype highlight the complex interplay between genetic and environmental factors in the pathogenesis of PE.

Earlier investigations have established a connection between the rs4343 genetic variant and a spectrum of health issues, notably affecting the heart and vascular system, such as enlargement of the left ventricle, elevated blood pressure levels, disorders of the coronary arteries, as well as migraine headaches [24,25,26,27]. These findings suggest that this particular polymorphism plays a significant role in the pathophysiology of both cardiovascular and cerebrovascular conditions, indicating its potential impact on the structural and functional integrity of the heart and blood vessels [27], along with contributing to the neurological manifestations observed in migraine sufferers [28].

The ACE G2350A polymorphism leads to a synonymous substitution of Thr 776 Thr, highlighting recent findings that even silent mutations, previously thought to be inconsequential, might indeed play a significant role in altering physiological functions, thereby potentially increasing the risk of developing diseases in humans [29]. At the level of messenger RNA (mRNA), such mutations have the potential to impact the molecule’s stability, how it folds, and the speed at which it is translated into proteins. Furthermore, these genetic alterations can lead to irregular splicing of the mRNA. Any changes to the mRNA can have significant consequences on the corresponding protein’s structure, how it is produced, its specificity for certain substrates, how it is secreted, and its enzymatic functions [28]. The primary way in which a silent mutation can alter the characteristics of a protein lies in the phenomenon known as codon usage bias. This concept describes how during the translation of mRNA into protein, certain codons are favored over their synonymous counterparts. The preference for specific codons over others can differ across various species, tissues, and even within individual genes, influencing how proteins are ultimately formed and function [30]. In 2018, Abedin and his team investigated how the rs4343 polymorphism influences the structure of mRNA and the speed of protein synthesis at a local level. Their research revealed that this particular genetic variation leads to a reduced speed of translation elongation for a rare codon when compared to its normal counterpart, which could, in turn, modify the characteristics of the enzyme produced. Additionally, it has been proposed that rs4343 might change the regulatory sequences for various transcription factors, potentially impacting the concentration of the ACE enzyme in the body [31].

Furthermore, the demographic and clinical characteristics of our study population, including factors such as BMI, marital status, and employment status, provide a comprehensive backdrop against which the genetic findings can be interpreted. The significant association of higher BMI with the PE group, for instance, aligns with existing literature on the role of obesity in increasing PE risk [32,33]. Similarly, the differences in employment status point towards socio-economic factors as potential contributors to PE risk [34,35], suggesting that PE is a multifaceted condition influenced by a multitude of genetic, environmental, and social factors [23].

The ACE G2350A polymorphism has been studied for its potential connection to PE. Research indicates that certain alleles of the G2350A polymorphism may be associated with increased ACE levels, leading to heightened vascular resistance and hypertension, both of which are key factors in the pathogenesis of PE. Studies have shown that pregnant women carrying the risk allele may have a higher predisposition to developing PE, suggesting that the ACE G2350A polymorphism could contribute to the genetic predisposition of this condition [36].

In light of these findings, our study contributes to the growing body of evidence on the genetic basis of PE and highlights the importance of considering a broad range of factors in understanding and managing this complex condition. The identification of genetic markers associated with PE risk not only enhances our understanding of the disease’s etiology but also offers potential pathways for early detection, prevention, and personalized treatment strategies. Future research should focus on expanding the genetic analysis to include a wider range of polymorphisms and exploring the interactions between genetic predisposition and environmental or lifestyle factors in the development of PE.

Recent studies underline the connection between preeclampsia and the increased risk of postpartum depression, highlighting the importance of careful monitoring and appropriate interventions during the perinatal period. Last but not least, early detection of preeclampsia is crucial in preventing subsequent complications, including postpartum depression, thus reinforcing a holistic approach to maternal-fetal health management [37].

### Strengths and Limitations

A significant strength lies in its comprehensive inclusion and exclusion criteria, which meticulously select participants to eliminate potential biases, thereby enhancing the reliability of the findings. The study’s setting in the “Pius Brînzeu” Emergency County Clinical Hospital in Timișoara, Romania, provides a unique demographic and clinical context, enriching the global understanding of PE. The detailed demographic and clinical profiling of the participants further strengthens the study by allowing for an integrated analysis of how genetic and various risk factors interplay in the development of PE. Collectively, these elements underscore the study’s contribution to advancing the understanding of PE’s genetic risk factors, laying the groundwork for future research and potential clinical applications in screening, prevention, and personalized treatment strategies.

This study, while offering valuable insights into the genetic underpinnings of PE, has several limitations that warrant consideration. Firstly, the case-control design, although effective for identifying associations, does not establish causality between genetic polymorphisms and PE risk. Secondly, the study is constrained by its sample size, which, while adequate for initial exploration, may limit the generalizability of the findings to broader populations. Additionally, the research focuses on a specific genetic polymorphism, potentially overlooking the multifactorial nature of PE that involves a complex interplay of multiple genetic and environmental factors. 

## 5. Conclusions

The study identified that PE is more frequent in women with the AA genotype compared to those with GG or AG genotypes. This significant finding highlights the role of specific genetic polymorphisms in the development of PE. The association with the AG genotype suggests potential pathways for future research into targeted interventions or screening strategies. The study highlights the importance of genetic factors in PE and the need for further investigation to develop targeted screening, prevention, and treatment strategies.

## Figures and Tables

**Table 1:** Demographic characteristics of participants by group.

Demographic Criteria	Control Group (N = 140)	PE Group (N = 128)	*p* Value
**Age** ^(a)^	30.16 ± 5.716	31.59 ± 6.843	0.0638
**Hospitalization days** ^(a)^	3.4 ± 6.0	5.0 ± 7.1	0.001
**BMI (kg/m^2^)** ^(b)^			
<25	112 (80%)	57 (44.53%)	<0.0001
25–29.9	19 (13.57%)	36 (28.12%)	0.0033
≥30	9 (6.42%)	35 (27.34%)	<0.0001
**Gravidity** ^(b)^			
Primigravida	52 (37.14%)	47 (36.71%)	0.9420
Multigravida	88 (62.85%)	81 (63.28%)	0.9420
**Marital Status** ^(b)^			
Married	83 (59.28%)	91(71.09%)	0.0434
Engaged	39 (27.85%)	22 (17.18%)	0.0378
In a relationship	13 (9.28%)	12 (9.37%)	0.9798
Single	5 (3.57%)	3 (2.34%)	0.5551
**Education** ^(b)^			
No formal education	5 (3.57%)	7 (5.46%)	0.4555
Primary education	11 (7.85%)	13 (10.15%)	0.5108
High school	67 (47.85%)	63 (49.21%)	0.8242
Higher education	59 (42.14%)	45 (35.15%)	0.2417
**Occupation** ^(b)^			
Unemployed	56 (40%)	71 (55.46%)	0.0115
Student	7 (5%)	3 (2.34%)	0.2519
Employed	77 (55%)	54 (42.18%)	0.0363
**Area of residence** ^(b)^			
Urban	69 (49.28%)	76 (59.37%)	0.0984
Rural	71 (50.71%)	52 (40.62%)	0.0984

PE—Preeclampsia; BMI—Body Mass Index; ^(a)^ mean ± std. dev.; ^(b)^ observed frequency (percentage).

**Table 2:** Vital Parametersupon hospital admission.

	Control Group	PE Group	*p* Value
SBP * mmHg Mean ± SD	112.6 ±12.06	165.9 ± 13.87	<0.0001
DBP ** mmHg Mean ± SD	76.25 ± 9.317	96.34 ± 9.010	<0.0001
Heart rate b/min Mean ± SD	84.96 ± 8.169	93.94 ± 7.817	<0.0001
SpO2 ***% Mean ± SD	98.61 ± 0.6198	98.25 ± 1.004	0.0004

* systolic blood pressure; ** diastolic blood pressure; *** oxygen saturation.

**Table 3:** Evaluation of the Differences in Apgar Scores, Gestational Age, and Neonatal Birth Weight between the Control Group and the PE Group.

Parameters	Control Group	PE Group	*p* Value
**Apgar Score Range (Mean ± SD)**	8.94 ± 0.60	8.55 ± 0.68	0.0055
**Gestation Duration (weeks)** **(Mean ± SD)**	38.38 ± 1.02	37.75 ± 2.24	0.0471
**Newborn Weight (grams) (Mean ± Standard Deviation)**	3363 ± 457	3118 ± 630	0.0392

**Table 4:** Genetic polymorphism RS4343 distribution and its association with PE risk.

Polymorphism	Control Group	PE Group	OR (95% CI)	*p* Value
GG	40 (28.57)	57 (44.53%)	Reference 0.498 (0.300–0.826) 1.638 (0.821–3.268)	0.021
AG	75 (53.57%)	56 (43.75%)		
AA	25 (17.85%)	15 (11.71%)		

## Data Availability

This data can be found here: www.umft.ro.
